# Predictors of futile recanalization following endovascular therapy for acute anterior circulation large vessel occlusion stroke: a retrospective cohort study

**DOI:** 10.3389/fneur.2026.1777164

**Published:** 2026-09-30

**Authors:** Yangchun Wen, Xi Liu, Xiujing Liao, Guanghong Zhong, Hongli Gu, Jia Zhang, Ying Xie

**Affiliations:** 1 Heyuan People’s Hospital, Heyuan, Guangdong, China; 2 Department of Neurology, Heyuan Hospital of Traditional Chinese Medicine, Heyuan, China; 3 Heyuan Key Laboratory of Molecular Diagnosis and Disease Prevention and Treatment, Doctors Station of Guangdong Province, Heyuan People's Hospital, Heyuan, Guangdong, China

**Keywords:** anterior circulation occlusion, cerebral infarction, endovascular therapy, futile recanalization, predictors

## Abstract

**Background:**

Endovascular therapy (EVT) is effective for acute anterior circulation large vessel occlusion (LVO), yet futile recanalization remains common. Identifying its predictors is essential to optimize patient selection.

**Methods:**

This retrospective cohort study included 311 patients with anterior circulation LVO who underwent successful EVT. Patients were divided into an effective recanalization group (90-day modified Rankin Scale (mRS) score ≤ 2, *n* = 150) and a futile recanalization group (mRS > 2, *n* = 161). Clinical, imaging, and procedural variables were compared. Multivariate logistic regression was used to identify independent predictors, and a predictive model was evaluated using receiver operating characteristic (ROC) curve analysis.

**Results:**

Baseline comparisons between the two groups showed significant differences in age, sex, atrial fibrillation, stress hyperglycemia ratio (SHR), C-reactive protein, admission National Institutes of Health Stroke Scale (NIHSS) score, diffusion-weighted imaging (DWI) infarct volume, DWI-Alberta Stroke Program Early CT Score (DWI-ASPECTS) score, number of procedure attempts, and procedure time. Univariate logistic regression and multivariate logistic regression identified admission NIHSS score (OR = 1.381, 95% CI: 1.113–1.713, *p* = 0.003), DWI infarct volume (OR = 1.057, 95% CI: 1.025–1.090, *p* < 0.001), DWI-ASPECT score (OR = 69.240, 95% CI: 9.819–488.254, *p* < 0.001), procedure time (OR = 1.011, 95% CI: 1.005–1.018, *p* < 0.001), and SHR (OR = 3.447, 95% CI: 1.302–9.129, *p* = 0.013) as independent predictors of futile recanalization. A combined model incorporating these five predictors achieved an area under the curve (AUC) of 0.815 (sensitivity: 0.789, specificity: 0.787, Youden index: 0.576).

**Conclusion:**

Admission NIHSS score, SHR, DWI infarct volume, DWI-ASPECT score, and procedure time are independent predictors of futile recanalization after EVT for anterior circulation LVO stroke. Higher NIHSS score, elevated SHR, larger infarct volume, lower DWI-ASPECT score, and prolonged procedure time increase the risk of poor functional outcome despite successful reperfusion.

## 1 Introduction

Acute cerebral infarction resulting from large anterior circulation artery occlusion accounts for approximately 30% of all ischemic strokes. This type of stroke represents a major cause of severe disability and mortality worldwide (1–3). Following several multicenter randomized controlled trials (RCTs) (4–8), EVT has been established as an effective treatment for acute cerebral infarction due to anterior circulation large artery occlusion and has been incorporated into major international guidelines. These guidelines strongly recommend the prompt administration of EVT to reduce mortality and disability in eligible patients (9). However, despite the extension of the treatment window to 24 h, futile recanalization persists in 30–60% of cases (4, 8, 10).

Futile recanalization following endovascular therapy for anterior circulation cerebral infarction is defined as a failure to achieve functional independence (mRS score >2 at 90 days) despite successful angiographic reperfusion (modified Thrombolysis in Cerebral Infarction (mTICI) grade of ≥2b) (11–13). The underlying pathological mechanism remains incompletely understood but may involve reperfusion injury and distal microemboli (14, 15).

Although the clinical significance of futile recanalization is recognized, its specific determinants remain unclear. This study aimed to identify factors associated with futile recanalization by retrospectively analyzing data from patients who underwent EVT for anterior circulation large artery occlusion at our center.

## 2 Methods

### 2.1 Study population

This retrospective study analyzed data from all patients presenting with cerebral infarction due to anterior circulation large artery occlusion who underwent endovascular therapy at Heyuan People’s Hospital from January 1, 2018 to December 31, 2023.

Patient inclusion required the following criteria: (1) age ≥18 years; (2) confirmation of occlusion by computed tomography angiography (CTA), magnetic resonance angiography (MRA), or digital subtraction angiography (DSA), with clinical symptoms attributable to anterior circulation occlusion; (3) successful reperfusion post-procedure, defined as mTICI grade of ≥2b; and (4) preoperative brain magnetic resonance imaging (MRI) with complete imaging data was available.

The exclusion criteria were: (1) unavailability of baseline mRS score; (2) inability to complete at least a three-month follow-up; (3) presence of any other condition causing severe neurological deficits, such as Parkinson’s disease/peripheral nerve disease, excluding anterior circulation large vessel occlusion stroke.

### 2.2 Time metrics

The onset time was determined according to guideline definitions (9). For patients with witnessed symptom onset, the time of the first symptom occurrence was recorded as the onset time. For those with unwitnessed onset, the time the patient was last known to be well was used. In cases where secondary neurological deterioration followed transient or mild symptoms, the time of this deterioration was considered the onset time of cerebral infarction.

### 2.3 Treatment procedures

Endovascular interventions for acute anterior circulation large artery occlusion encompassed a range of techniques, including balloon angioplasty, stent placement, stent retriever thrombectomy, aspiration thrombectomy, and intra-arterial thrombolysis, all performed under cerebral angiography guidance. The specific treatment strategy was determined by the operating surgeon based on the individual patient’s clinical presentation and angiographic findings. Local anesthesia was the preferred approach, while general anesthesia or a combination of both was selected for specific cases at the discretion of the surgical and anesthesia teams. All enrolled patients received standardized clinical management and care during hospitalization and follow-up.

### 2.4 Neuroimaging evaluation

The mTICI score following the intervention was assessed jointly by the primary operating surgeon and the first assistant. The mTICI grading system was defined as follows: grade 0, indicating no distal flow; grade 1, penetration with minimal perfusion; grade 2a, partial reperfusion of 1–49% of the target territory; grade 2b, 50–89% reperfusion; grade 2c, 90–99% reperfusion; and grade 3, complete (100%) reperfusion.

Preoperative infarct extent was quantified using the ASPECTS on baseline imaging by two experienced neuroradiologists. The ASPECTS was evaluated on the preoperative magnetic resonance diffusion-weighted imaging (MR-DWI) sequence. This 10-point topographic scoring system divides the middle cerebral artery (MCA) territory into 10 regions across two levels. One point is subtracted from the initial score of 10 for each region showing an ischemic lesion. For the M1–M6 cortical regions, a point is deducted if the area of restricted diffusion occupies ≥1/3 of the region. The preoperative infarct volume was semi-automatically segmented on the MR-DWI sequence (16), using the artificial intelligence software. This software, the CTPoc AI software (SHUKUN, Beijing, China), was originally developed for computed tomography (CT) / CT perfusion (CTP)-based volume assessment (17), and has now been extended to MR sequences for volumetric measurement using analogous algorithms. The preoperative DWI infarct volume was independently evaluated by two experienced neuroradiologists. Interobserver reliability for the preoperative DWI infarct volume measurements was evaluated using the intraclass correlation coefficient (ICC), whereas that for the final DWI-ASPECTS scores was determined using the Kappa coefficient (18).

### 2.5 Data collection

Patient demographic and clinical characteristics were systematically collected. These included: (a) Characteristics and clinical features: gender, age, and medical history, including hypertension, coronary heart disease, rheumatic heart disease, atrial fibrillation, history of endotracheal intubation, SHR, blood uric acid, total cholesterol, high-density lipoprotein (HDL) cholesterol, low-density lipoprotein (LDL) cholesterol, C-reactive protein, homocysteine, time from symptom onset, serum creatinine, fibrinogen, systemic immune-inflammation index (SII), admission NIHSS score, mRS score, infarct area on DWI sequence, and ASPECTS on DWI; (b) Operative characteristics: procedure time and number of endovascular intervention attempts.

### 2.6 Outcome assessment and group stratification

Functional outcome was assessed 90 (±14) days post-procedure using the mRS score via face-to-face or structured telephone interviews. Based on the 90-day mRS score, enrolled patients were stratified into two groups: the effective recanalization group (mRS ≤ 2), indicating functional independence; and the futile recanalization group (mRS > 2), representing poor functional outcome.

### 2.7 Statistical analysis

Statistical analyses were performed using SPSS software (version 22.0). Missing data in the dataset was addressed using multiple imputations. Categorical data are presented as numbers and percentages and were compared between the two groups using the Chi-square test. Continuous data were first assessed for normality and homogeneity of variance. Data conforming to a normal distribution are expressed as the mean ± standard deviation (SD) and were compared using the independent-samples t-test. Non-normally distributed data are summarized as the median and interquartile range and were compared using the Mann–Whitney U test. The primary endpoint for both groups was futile recanalization (defined as a 3-month mRS score > 2). Factors showing statistical significance between groups were entered into univariate logistic regression. Those with significance in the univariate analysis were then included in a multivariate model to identify independent predictors. ROC curves were used to evaluate the discriminatory ability of independent factors, and sensitivity and specificity were derived. All tests were two-sided, with *p* < 0.05 considered significant.

## 3 Results

A total of 311 patients with acute cerebral infarction resulting from large anterior circulation artery occlusion who underwent endovascular therapy were included in this study, with the enrollment process detailed in Figure 1. Based on the 90-day mRS scores, patients were classified into two groups: the effective recanalization group (mRS ≤ 2, *n* = 150) and the futile recanalization group (mRS > 2, *n* = 161). The comparison of baseline, preoperative, intraoperative, and postoperative characteristics between the two groups is detailed in Table 1.

**Figure 1:**
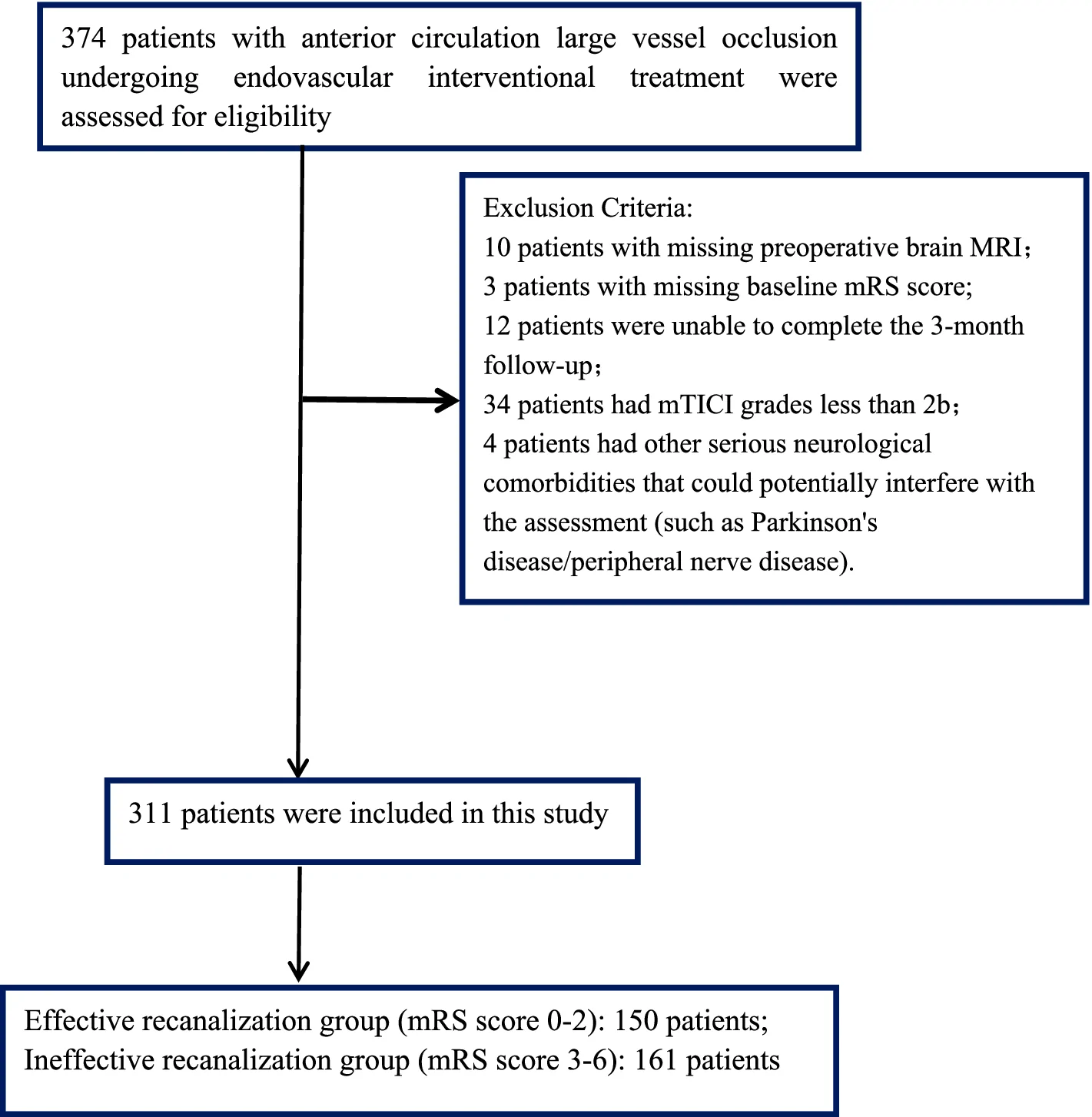
The enrollment diagram for patients with acute anterior circulation large vessel occlusion undergoing endovascular intervention.

**Table 1:** Comparison of patient demographic and clinical characteristics between the effective recanalization and futile recanalization groups.

Variable(effective sample size)	Effective recanalization (*n* = 150)	Futile recanalization (*n* = 161)	Test statistic	*p*-value
Age (years)	64(57.00, 72.00)	67(58.00, 76.00)	2.263	0.009^b^
Female	37(24.7)	66(40.9)	9.345	0.002^a^
History of Hypertension	79(52.7)	92(57.1)	0.629	0.249^a^
History of Coronary Heart Disease	11(7.3)	20(12.4)	2.241	0.095^a^
History of Rheumatic Heart Disease	3(2.0)	7(4.3)	1.375	0.198^a^
Pre-onset mRS score			0.212	0.388^a^
0 points	133	140		
1 points	17	21		
Atrial Fibrillation	16(10.7)	30(18.6)	3.911	0.034^a^
SHR	0.89(0.74, 1.08)	1.08(0.90, 1.37)	4.177	<0.001^b^
Serum Uric Acid (μmol/L)	310.00(248.00, 381.25)	296.00(241.00, 363.50)	0.998	0.252^b^
Serum Creatinine (μmol/L)	77.00(64.00, 92.00)	77.00(63.00, 94.00)	0.123	0.888^b^
Total Cholesterol (mmol/L)	4.47(4.01, 4.97)	4.64(3.68, 5.23)	0.397	0.648^b^
HDL Cholesterol (mmol/L)	1.13(0.99, 1.35)	1.18(1.02, 1.43)	1.260	0.148^b^
LDL Cholesterol (mmol/L)	2.62(2.18, 3.07)	2.55(1.99, 3.10)	0.789	0.365^b^
Fibrinogen (g/L)	3.24(2.73, 3.73)	3.26(2.76, 3.78)	0.272	0.755^b^
Systemic Immune-Inflammation Index	808.00(519.50, 1639.75)	1025.00(593.5.00, 1702.50)	1.106	0.204^b^
Homocysteine (μmol/L)	11.00(8.00, 15.00)	10.70(7.00, 14.50)	1.496	0.086^b^
C-reactive Protein (mg/L)	0.50(0.50, 1.15)	0.70(0.50, 2.00)	3.174	<0.001^b^
Admission NIHSS Score	11.00(8.00, 15.00)	14.00(11.00, 17.00)	6.488	<0.001^b^
Intravenous thrombolysis	58(38.7)	68(42.2)	0.411	0.300^a^
Obstructed area			1.113	0.774^a^
Internal carotid artery	28	26		
Middle cerebral artery M1	61	72		
Middle cerebral artery M2	24	21		
Tandem lesions	37	42		
mTICI grading			0.228	0.360^a^
2b	52	60		
3	98	101		
Onset to puncture time (minutes)	430.00(265.00, 688.25)	421.00(273.50, 606.00)	0.886	0.309^b^
TOAST Classification			5.313	0.065^a^
Large artery atherosclerosis	121(80.7)	119(73.9)		
Cardioembolism	25(16.7)	41(25.5)		
Undetermined etiology	4(2.7)	1(0.6)		
Number of procedure attempts	2.38(1.20)	2.89(1.45)	−3.754	<0.001^c^
Procedure time (minutes)	96.79(42.05)	110.55(50.03)	−3.313	0.004^c^
DWI infarct volume (ml)	37.60(16.95)	57.62(25.49)	5.429	<0.001^c^
DWI-ASPECT score	6.55(1.28)	5.89(1.11)	17.891	<0.001^c^
3–5	41	56		
6–7	73	94		
8–10	36	11		

Superscript letters denote the statistical test used for group comparisons in the corresponding column.

a Chi-square test; values are expressed as n(%).

b Mann–Whitney U test; values are expressed as median (Q1, Q3).

c Independent samples *t*-test; values are expressed as mean (SD).

Systemic Immune-Inflammation Index (SII) = (Platelet count × Neutrophil count)/Lymphocyte count. P: platelet count in complete blood count; N: neutrophil count in complete blood count; L: lymphocyte count in complete blood count.

Stress Hyperglycemia Ratio (SHR) = Admission glucose (mmol/L) / [1.59 × HbA1c (%) – 2.59]. Admission glucose: fasting plasma glucose or random blood glucose at admission; HbA1c: glycated hemoglobin.

Missing data in the dataset was addressed using multiple imputations.

### 3.1 Patient demographic and clinical characteristics

The effective recanalization group comprised 150 patients, including 37 females (24.7%) and 113 males, with a median age of 64 (57.00, 72.00) years. In this group, Atrial fibrillation was present in 16 patients (10.7%), and the SHR was 0.89 (0.74, 1.08). The median C-reactive protein level was 0.50 (0.50, 1.15) mg/L, and the median admission NIHSS score was 11.00 (8.00, 15.00). The mean number of procedure attempts was 2.38 (1.20), the mean procedure time was 96.79 (42.05) minutes, the mean DWI infarct volume was 37.60 (16.95) mL, and the mean DWI-ASPECT score was 6.55 (1.28).

The futile recanalization group comprised 161 patients, including 66 females (40.9%) and 95 males, with a median age of 67 (58.00, 76.00) years. In this group, Atrial fibrillation was present in 30 patients (18.6), the SHR was 1.08(0.90, 1.37). The median C-reactive protein was 0.70 (0.50, 2.00) mg/L, and the median admission NIHSS score was 14.00 (11.00, 17.00). The mean number of procedure attempts was 2.89(1.45), and the mean procedure time was 110.55(50.03) min, the mean DWI infarct volume was 57.62(25.49) mL, and the mean DWI-ASPECT score was 5.89(1.11).

Between the two groups, there were significant differences in age (*p* = 0.009), sex (*p* = 0.002), atrial fibrillation (*p* = 0.034), SHR (*p* < 0.001), C-reactive protein (*p* < 0.001), admission NIHSS score (*p* < 0.001), number of procedure attempts (*p* < 0.001), procedure time (*p* = 0.004), DWI infarct volume (*p* < 0.001), and DWI-ASPECT score (*p* < 0.001), with interobserver reliability for DWI infarct volume being excellent (ICC = 0.92, 95% CI: 0.91–0.95) and for DWI-ASPECTS showing good agreement (weighted Kappa = 0.77, 95% CI: 0.72–0.84). There were no significant differences between the two groups in terms of history of hypertension, history of coronary heart disease, history of rheumatic heart disease, pre-onset mRS score, serum uric acid, serum creatinine, total cholesterol, HDL cholesterol, LDL cholesterol, fibrinogen, systemic immune-inflammation index, homocysteine, intravenous thrombolysis, Obstructed area, mTICI grading, onset to puncture time, or Trial of Org 10,172 in Acute Stroke Treatment (TOAST) classification.

### 3.2 Logistic regression analysis of factors associated with futile recanalization

Univariate logistic regression was performed on factors showing significant between-group differences. Variables significant in the univariate analysis were then included in a multivariate model to identify independent predictors, and the results are summarized in Table 2.

**Table 2:** Univariate and multivariate logistic regression analyses of factors associated with futile recanalization.

Clinical characteristics	Univariate analysis			Multivariate analysis		
	OR	95% CI	*p* value	OR	95% CI	*p* value
Gender	1.630	0.848–3.131	0.142			
Age	0.990	0.965–1.015	0.431			
Atrial fibrillation	1.456	0.598–3.542	0.408			
Admission NIHSS score	1.414	1.134–1.761	0.002	1.381	1.113–1.713	0.003
DWI infarct volume	1.053	1.021–1.087	<0.001	1.057	1.025–1.090	<0.001
DWI-aspect score	93.914	12.623–698.740	<0.001	69.240	9.819–488.254	<0.001
Number of procedure attempts	1.264	0.983–1.624	0.068			
Procedure time	1.009	1.001–1.016	0.024	1.011	1.005–1.018	<0.001
SHR	2.949	1.104–7.878	0.031	3.447	1.302–9.129	0.013
C-reactive protein	1.095	0.930–1.290	0.276			

In the univariate analysis, the admission NIHSS score(OR = 1.414, 95% CI: 1.134–1.761, *p* = 0.002), DWI infarct volume (OR = 1.053, 95% CI: 1.021–1.087, *p* < 0.001), DWI-ASPECT score (OR = 93.914, 95% CI: 12.623–698.740, *p* < 0.001), procedure time (OR = 1.009, 95% CI: 1.001–1.016, *p* = 0.024), and SHR (OR = 2.949, 95% CI: 1.104–7.878, *p* = 0.031) were significantly associated with futile recanalization. Gender, age, atrial fibrillation, number of procedure attempts, and C-reactive protein did not reach statistical significance in the univariate analysis.

In the multivariate analysis, the admission NIHSS score(OR = 1.381, 95% CI: 1.113–1.713, *p* = 0.003), DWI infarct volume (OR = 1.057, 95% CI: 1.025–1.090, *p* < 0.001), DWI-ASPECT score (OR = 69.240, 95% CI: 9.819–488.254, *p* < 0.001), procedure time (OR = 1.011, 95% CI: 1.005–1.018, *p* < 0.001), and SHR (OR = 3.447, 95% CI: 1.302–9.129, *p* = 0.013) remained independent predictors of futile recanalization.

### 3.3 Predictive performance of the model

The discriminatory ability of individual predictors and the combined model for distinguishing futile recanalization was evaluated using ROC curve analysis, as shown in Figure 2. The admission NIHSS score yielded an AUC of 0.733, with a sensitivity of 0.733 and a specificity of 0.560. The SHR model produced an AUC of 0.610, with a sensitivity of 0.634 and a specificity of 0.533. The DWI infarct volume model demonstrated an AUC of 0.743, with a sensitivity of 0.745 and a specificity of 0.647. The DWI-ASPECT score model showed an AUC of 0.591, with a sensitivity of 0.348 and a specificity of 0.727. The procedure time model yielded an AUC of 0.581, with a sensitivity of 0.621 and a specificity of 0.513. The combined prognostic model, incorporating all significant predictors, achieved an AUC of 0.815, with a sensitivity of 0.789, a specificity of 0.787, and a Youden index of 0.576.

**Figure 2:**
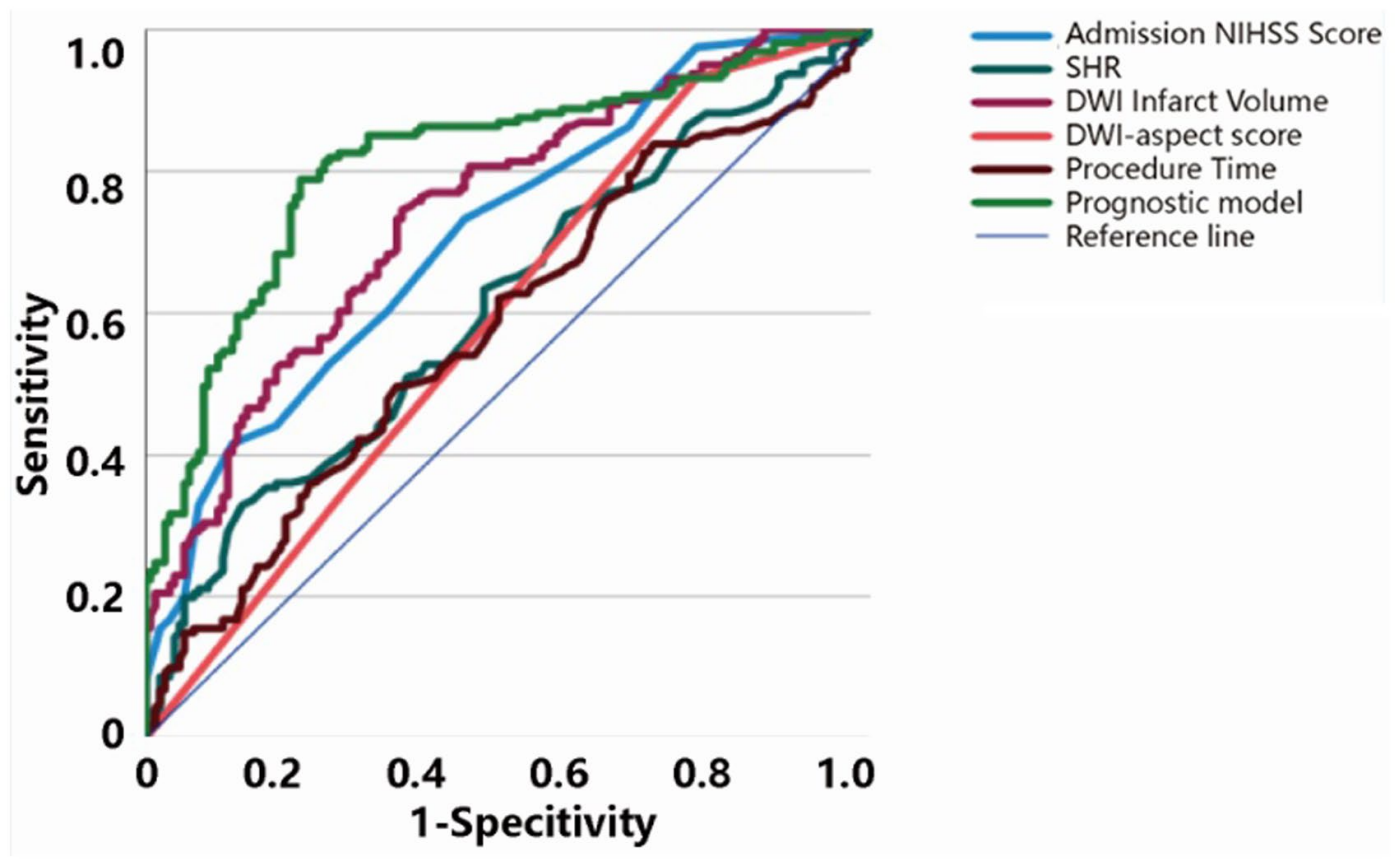
Ability of a combination of predictors to distinguish futile recanalization. The area under the curve for the Admission NIHSS score was 0.733, with a sensitivity of 0.733 and specificity of 0.56. The area under the curve for the SHR was 0.610, with a sensitivity of 0.634 and specificity of 0.533. The area under the curve for the DWI infarct volume was 0.743, with a sensitivity of 0.745 and specificity of 0.647. The area under the curve for DWI-ASPECT score was 0.591, with a sensitivity of 0.348, specificity of 0.727. The area under the curve for the Procedure time was 0.581, with a sensitivity of 0.621, specificity of 0.513. The area under the curve for the Prognostic model was 0.815, with a sensitivity of 0.789, specificity of 0.787, and a Youden index of 0.576.

## 4 Discussion

Although EVT has been established as a highly effective treatment for acute anterior circulation large vessel occlusion stroke through numerous randomized controlled trials, futile recanalization remains a persistent clinical challenge, affecting up to 30–60% of successfully recanalized patients (8). The phenomenon of futile recanalization represents a critical disconnect between successful revascularization and meaningful functional recovery, underscoring the need to identify its underlying risk factors. Therefore, in this retrospective cohort study, we aimed to investigate the factors influencing futile recanalization in patients with anterior circulation large vessel occlusion who underwent EVT. Our findings revealed that admission NIHSS score, SHR, DWI infarct volume, DWI-ASPECT score, and procedure time were independent predictors of futile recanalization. Furthermore, a predictive model combining these three factors demonstrated robust discriminative ability, with an AUC of 0.815, suggesting its potential clinical utility in risk stratification.

In our multivariate model, the admission NIHSS score was also an independent predictor of futile recanalization (OR = 1.381, *p* = 0.003). The NIHSS is a well-validated measure of baseline stroke severity and has been consistently associated with functional outcomes in numerous stroke studies (19, 20). Previous studies have shown that higher admission NIHSS scores reflect more severe neurological deficits, which may limit the potential for meaningful recovery even after successful recanalization. Our findings reinforce the NIHSS as a robust predictor of futile recanalization in patients with anterior circulation large artery occlusion undergoing endovascular therapy, underscoring its clinical utility as an easily accessible tool for early risk stratification.

The higher SHR at admission emerged as another independent predictor of futile recanalization. This finding is consistent with a growing body of evidence linking stress hyperglycemia to worse outcomes in acute ischemic stroke (21). Unlike absolute glucose values, SHR accounts for baseline glycemic status by incorporating glycated hemoglobin (HbA1c), thereby providing a more accurate reflection of the relative increase in blood glucose during acute stress rather than merely representing absolute hyperglycemia (22). The deleterious effects of hyperglycemia are likely mediated through multiple mechanisms, including enhanced oxidative stress, promotion of inflammatory responses, disruption of the blood–brain barrier, and impaired cerebral microcirculatory reperfusion (23). In the setting of EVT, hyperglycemia may amplify reperfusion injury, thereby diminishing the net clinical benefit of recanalization. As a comprehensive indicator of stress-induced hyperglycemia, SHR integrates admission glucose with baseline glycemic status and may offer superior prognostic value over admission glucose alone.

DWI infarct volume was identified as a robust independent predictor of futile recanalization. This finding aligns with the well-established pathophysiological concept that the volume of the ischemic core at baseline is a critical determinant of eventual functional outcomes (24–26). A larger DWI infarct volume signifies a greater extent of irreversible neuronal damage prior to reperfusion, thereby limiting the potential benefit of any revascularization attempt. Even with successful recanalization, the substantial loss of brain parenchyma may preclude functional independence. This is consistent with our observation that the effective recanalization group had a significantly smaller mean infarct volume (37.60 mL) compared to the futile recanalization group (57.62 mL). From a clinical perspective, this finding underscores that successful recanalization does not equate to tissue salvage when the ischemic core is already extensive. Thus, DWI infarct volume may serve as a crucial imaging biomarker for patient selection, helping clinicians identify those most likely to benefit from EVT.

DWI-ASPECT score also emerged as an independent predictor of futile recanalization in our multivariate analysis, with a notably large odds ratio (OR = 69.240). The ASPECTS is a semi-quantitative topographic scoring system that provides a rapid, practical assessment of early ischemic changes (27). A lower DWI-ASPECT score reflects more widespread involvement of the MCA territory, which inherently correlates with larger infarct volume and greater baseline neurological severity (28). The strong association between DWI-ASPECT score and futile recanalization observed in our study reinforces its clinical utility as a bedside tool for prognostication.

Procedure time was also identified as an independent predictor of futile recanalization. Longer procedure times were associated with a significantly increased risk of poor functional outcomes, even after successful recanalization. This association can be attributed to several interrelated factors. Prolonged endovascular manipulation may increase the risk of periprocedural complications, including distal embolization, vessel perforation, and reperfusion injury. Furthermore, extended procedure time may reflect technical difficulty, such as challenging vascular anatomy or resistant thrombus, which can prolong the duration of cerebral hypoperfusion and exacerbate ischemic injury (29). Although the total time from onset to recanalization is a well-recognized prognostic factor, our study highlights the procedural duration itself as a critical and potentially modifiable factor. Efforts to streamline the procedure and achieve rapid recanalization, without compromising safety, may help reduce the likelihood of futile recanalization.

In addition to the independent predictors identified, our study also revealed significant differences between the two groups in several other baseline characteristics, including age, sex, atrial fibrillation, C-reactive protein levels, and number of procedure attempts. These factors did not retain statistical significance in the multivariate model. However, their presence in the univariate analysis suggests potential associations that warrant further investigation in larger, prospective studies. For instance, the observed differences in age and sex may reflect variations in vascular anatomy, collateral status, or underlying stroke mechanisms, all of which could influence the response to EVT (30, 31).

This study has several limitations that should be acknowledged. First, as a single-center retrospective study, the findings may be subject to selection bias and may not be generalizable to other populations or healthcare settings. Second, the relatively modest sample size limited our ability to perform more granular subgroup analyses or to adjust for a wider array of potential confounders. Third, the angiographic records in our center were established before the widespread adoption of the mTICI 2c grading scheme. Consequently, detailed data distinguishing mTICI 2c from 2b were not available in the original records, and these patients were collectively classified as mTICI 2b. Although this classification is unlikely to have affected the overall assessment of futile recanalization, it precludes further analysis of the potential impact of more refined reperfusion grades (particularly mTICI 2c vs. 2b) on futile recanalization outcomes. Fourth, the lack of perfusion imaging data, such as CT perfusion, precluded a more comprehensive assessment of the ischemic penumbra, which could have provided additional insights into the interplay between core volume, salvageable tissue, and futile recanalization. Fifth, due to regional characteristics or other potential factors, the proportion of large artery atherosclerosis (LAA) in the TOAST classification was notably high in our cohort, which may have introduced bias. Sixth, owing to the limited sample size, we found that inclusion of post-procedural variables such as pneumonia, vascular re-occlusion, and hemorrhagic transformation significantly influenced the statistical analysis of other factors; therefore, we did not incorporate these variables in the current study. This may have resulted in an incomplete assessment of factors associated with futile recanalization. Seventh, all enrolled patients underwent preoperative brain MRI, which may have inevitably prolonged the time to recanalization and potentially introduced bias in the assessment of outcomes, as the additional imaging time could have delayed the initiation of endovascular therapy.

## 5 Conclusion

In conclusion, this retrospective cohort study identified admission NIHSS score, SHR, DWI infarct volume, DWI-ASPECT score, and procedure time as independent predictors of futile recanalization following endovascular therapy in patients with acute anterior circulation large vessel occlusion stroke. Higher admission NIHSS score, elevated SHR, larger DWI infarct volume, lower DWI-ASPECT score, and prolonged procedure time were associated with an increased risk of futile recanalization. A combined predictive model incorporating these five factors demonstrated good discriminative ability, highlighting its potential clinical utility for early risk stratification. These findings may aid clinicians in identifying patients at high risk of futile recanalization, facilitating more personalized treatment decisions and optimized patient selection for EVT. However, further largescale, multicenter, prospective studies are warranted to validate these findings and to explore additional factors that may contribute to futile recanalization in this patient population.

## Data Availability

The raw data supporting the conclusions of this article will be made available by the authors, without undue reservation.
